# Design and SAR Analysis of an AMC-Integrated Wearable Cavity-Backed SIW Antenna

**DOI:** 10.3390/mi15121530

**Published:** 2024-12-23

**Authors:** Yathavi Thangavelu, Balakumaran Thangaraju, Rajagopal Maheswar

**Affiliations:** 1 Department of ECE, Coimbatore Institute of Technology, Coimbatore 641 014, India; balakumaran@cit.edu.in; 2 Department of ECE, Centre for IoT and AI (CITI), KPR Institute of Engineering and Technology, Coimbatore 641 407, India; maheshh3@rediffmail.com

**Keywords:** wearable antenna, substrate integrated waveguide, artificial magnetic conductor, specific absorption rate, textile antenna, wearable antenna

## Abstract

Wearable communication technologies necessitate antenna designs that harmonize ergonomic compatibility, reliable performance, and minimal interaction with human tissues. However, high specific absorption rate (SAR) levels, limited radiation efficiency, and challenges in integration with flexible materials have significantly constrained widespread deployment. To address these limitations, this manuscript introduces a novel wearable cavity-backed substrate-integrated waveguide (SIW) antenna augmented with artificial magnetic conductor (AMC) structures. The proposed architecture is meticulously engineered using diverse textile substrates, including cotton, jeans, and jute, to synergistically integrate SIW and AMC technologies, mitigating body-induced performance degradation while ensuring safety and high radiation efficiency. The proposed design demonstrates significant performance enhancements, achieving SAR reductions to 0.672 W/kg on the spine and 0.341 W/kg on the forelimb for the cotton substrate. Furthermore, the AMC-backed implementation attains ultra-low reflection coefficients, as low as −26.56 dB, alongside a gain improvement of up to 1.37 dB, culminating in a total gain of 7.09 dBi. The impedance bandwidth exceeds the ISM band specifications, spanning 150 MHz (2.3–2.45 GHz). The design maintains remarkable resilience and operational stability under varying conditions, including dynamic bending and proximity to human body models. By substantially suppressing back radiation, enhancing directional gain, and preserving impedance matching, the AMC integration optimally adapts the antenna to body-centric communication scenarios. This study uniquely investigates the dielectric and mechanical properties of textile substrates within the AMC-SIW configuration, emphasizing their practicality for wearable applications. This research sets a precedent for wearable antenna innovation, achieving an unprecedented balance of flexibility, safety, and electromagnetic performance while establishing a foundation for next-generation wearable systems.

## 1. Introduction

Wearable antennas are pivotal enablers of wireless body area networks (WBANs), where body-centric communication systems require compact, efficient, and safe designs that operate seamlessly close to the human body. The high dielectric properties of human tissues pose a critical challenge by shifting resonance frequencies, attenuating signals, and causing undesirable energy absorption, thereby degrading antenna performance. This makes the development of robust wearable antenna designs imperative. Overcoming these limitations involves the use of flexible materials, such as textiles (e.g., cotton, denim, and jute), flexible substrates (e.g., Kapton and polyimide), and other innovative materials like dielectric resonators and polydimethylsiloxane [1].

Among emerging technologies, substrate-integrated waveguide (SIW) antennas [2] have proven to be particularly advantageous due to their unique capability to combine compactness, low-loss electromagnetic wave propagation, and efficient impedance matching. By incorporating metallic vias into their dielectric substrates, SIW antennas confine and direct electromagnetic waves with precision, minimizing energy leakage and undesirable coupling effects [3]. This makes them superior to traditional patch antennas in wearable applications, where efficiency, compact design, and reliable performance are critical.

The integration of artificial magnetic conductor (AMC) surfaces with SIW technology provides an advanced solution to several performance challenges [4]. AMC surfaces act as electromagnetic reflectors, redirecting radiation away from the body, reducing back radiation, and significantly improving antenna gain and efficiency. Furthermore, their ability to suppress surface waves enhances the radiation pattern and mitigates the specific absorption rate (SAR), ensuring safety during prolonged use. The resulting design achieves improved performance across frequency bands operating across the 2.3–2.45 GHz ISM band, enabling its application in health monitoring, wireless communication, and IoT-driven wearable systems [5].

### 1.1. Related Works

Numerous studies have advanced wearable antenna design by incorporating innovative substrates and reflective structures to address performance constraints. These efforts include the development of compact dual-band antennas, textile-integrated patch antennas, and SIW cavity-backed configurations optimized for wearable applications [6]. Early research on wearable antennas focused on leveraging flexible textile substrates for improved conformability and integration into garments [7]. Materials such as denim, fleece, and felt have shown promise in maintaining mechanical flexibility while providing adequate dielectric properties. However, challenges such as higher loss tangents and reduced electromagnetic efficiency remain, particularly under human body interaction. Substrate-integrated waveguide (SIW) antennas, designed with planar waveguide structures, effectively confine electromagnetic energy and operate in the dominant transverse electric (TE_10_) mode [8,9,10]. These designs ensure efficient wave propagation while minimizing spurious radiation, making them ideal for high-frequency wearable applications. Despite their potential, many studies have been confined to rigid substrates like Rogers materials, limiting their suitability for flexible, body-worn applications. MC structures are increasingly employed in wearable antenna designs to mitigate back radiation, enhance gain, and reduce SAR levels. Amsaveni et al. demonstrated the integration of AMC reflectors to redirect electromagnetic waves away from the human body, ensuring minimal tissue absorption while maintaining efficient power transfer [11,12,13]. Additionally, the work by H. I. Azeez et al. highlighted how EBG and FSS structures can complement AMC designs for dual-band antennas [14].

Joshi et al. employed AMC backings to create localization-friendly antennas with dual-band capabilities for GPS and WBAN systems, achieving significant gains while adhering to safety standards like FCC and ICNIRP [15,16,17]. Similarly, Dwivedi et al. optimized the radiation patterns of UWB antennas using AMC-backed slot configurations, achieving superior performance metrics. Textiles such as cotton, denim, and jute have been extensively researched for wearable applications [18]. These materials offer flexibility, lightweight properties, and comfort, making them ideal for garment integration. Researchers like Alemaryeen et al. integrated AMC-backed textile antennas for WBAN systems, demonstrating enhanced impedance matching and bending resilience under dynamic conditions [19,20,21]. Zhang et al. explored metasurface-enhanced wearable antennas, emphasizing the role of flexible substrates in the achievement of lightweight designs with reduced radiation leakage. Smida et al. extended this approach, presenting broadband designs with applicability in biomedical telemetry [22].

### 1.2. Research Gap and Contribution

Previous designs focusing on rigid substrates like Rogers materials, while efficient, are unsuitable for wearable applications due to a lack of flexibility and conformability. Textile-based SIW cavity-backed antennas with AMC enhancements offer substantial improvements, including:

- lower SAR (e.g., 0.672 W/kg for spine using cotton substrate);
- higher gain and bandwidth compatibility;
- superior bending resilience.

The literature underscores a limited focus on the synergistic integration of AMC-backed SIW technology with textile substrates. Most existing research does not extensively evaluate real-world wearability parameters, such as dynamic bending or long-term proximity effects on human tissues.

### 1.3. Scope and Contribution

This manuscript introduces a novel wearable cavity-backed SIW antenna that incorporates AMC structures and leverages flexible textile substrates, specifically cotton, jeans, and jute. The proposed design bridges a critical research gap by:

- demonstrating a wearable antenna that maintains high-performance metrics, including a reflection coefficient as low as −26.56 dB and a gain improvement of 1.37 dB under human proximity and dynamic bending scenarios;
- achieving substantial SAR reduction (e.g., 0.672 W/kg on the spine and 0.341 W/kg on the forelimb) without compromising the impedance bandwidth;
- advancing the integration of AMC-backed SIW technology within textile substrates, ensuring comfort, adaptability, and safety in real-world wearable applications.

This study bridges the gap in textile-based, wearable SIW antenna designs, establishing benchmarks for future research in WBANs, IoT, and smart textile technologies.

## 2. Substrate-Integrated Waveguide (SIW) Cavity-Backed Antenna Design for Wearable Applications

**Figure 1:**
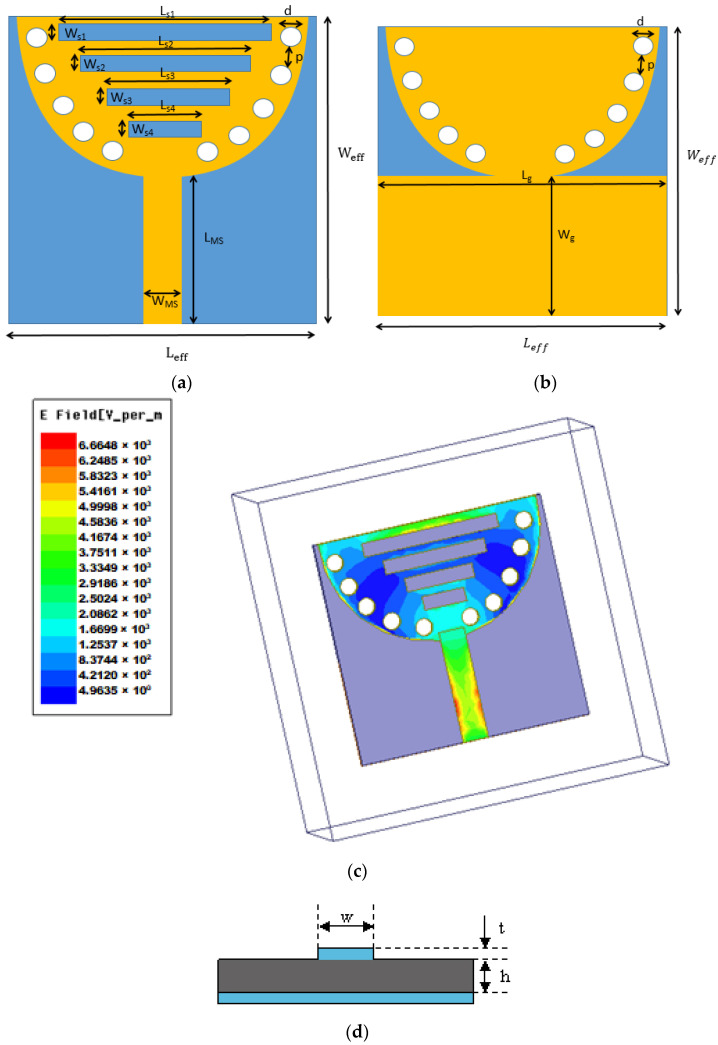
Geometry of cavity-backed SIW antenna on a wearable substrate showing (**a**) front-end view, (**b**) ground plane, (**c**) surface current distribution on the cavity-backed SIW antenna patch, and (**d**) microstrip feedline structure.

The substrate-integrated waveguide (SIW) cavity-backed antenna is designed with a radiating patch located on one side of a dielectric substrate and a ground plane on the opposite side. This configuration employs a textile-based substrate, such as cotton, denim (jean), or jute, reinforced with copper layers on its top and bottom surfaces to form the cavity structure. Metal vias along the substrate edges act as artificial sidewalls, ensuring the effective confinement of electromagnetic waves and facilitating the TE_10_ mode for efficient wave propagation. The antenna is excited using a microstrip feedline, which introduces energy into the cavity, while the radiating patch enables outward energy emission, as illustrated in Figure 1a.

**Figure 2:**
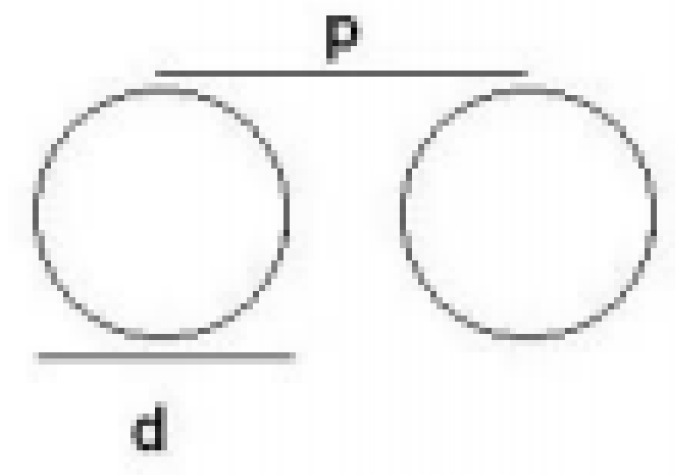
SIW vias structures.

In Figure 2, d is the diameter of metal vias, and p is the separation distance of the vias.

To minimize leakage that results from the distance between adjacent vias, it is essential to meet d ≤ λ_g_/5, p ≤ 2d. The variable λ_g_ represents the guided wavelength. The dimensions of the cavity-backed SIW textile antenna are derived from the following Equations (1)–(3) [23]:

(1) $$f=\frac{1}{2}\frac{c}{\sqrt{\varepsilon_{r}\mu_{r}}}\sqrt{{\left(\frac{1}{L_{eff}}\right)}^{2}+{\left(\frac{1}{W_{eff}}\right)}^{2}}$$

(2) $$L_{eff}=L+\frac{d^{2}}{10*L}-\frac{d^{2}}{0.95*p}$$

(3) $$W_{eff}=W+\frac{d^{2}}{10*W}-\frac{d^{2}}{0.95*p}$$

In Table 1, The physical dimensions of the substrate, specifically the length (L) and width (W), can be determined using the following relationships: L = λ_g_/(2√εr) and W = λ_g_/(2√(εr − 1)). Where ε_r_ is the substrate material’s relative permittivity, µ_r_ is the relative permeability of the substrate (not explicitly used in these equations), c is the velocity of light in a vacuum (approximately 3 × 10^8^ m/s), λ_g_ is the guide wavelength and should be λ_g_ = λ_0_/(√εr), and the λ_0_ wavelength in free space is calculated as λ_0_ = c/f. In the design of the cavity-backed SIW textile antenna [24], the effective dimensions are represented by L_eff_ (effective length) and W_eff_ (effective width). The ground plane length (L_g_) is designed to match the effective length of the substrate in length, while its width (W_g_) is set to half the effective width of the substrate. Four slots, along with a defective ground, are used to enhance the performance of the cavity-backed SIW patch antenna by changing the current distribution in the radiating patch.

The slot lengths (L_s1_, L_s2_, L_s3_, and L_s4_) are determined by the following relationships:

L_s1_ = L_eff_/2, L_s2_ = L_eff_/3, L_s3_ = L_eff_/4, L_s4_ = L_eff_/5

The widths of all slots are kept uniform and are calculated as follows:

W_s1_ = W_s2_ = W_s3_ = W_s4_ = W_eff_/8

The length of the microstrip feedline (L_MS_) can be calculated by λ_o_/5. The width of the microstrip feedline (W_MS_) feedline can be calculated by using Formula (4) [25].

(4) $$W_{Ms}=\frac{7.48\times h_{s}\;}{e^{\left(Z_{o}\frac{\sqrt{\varepsilon_{r}+1}}{87}\right)}}-1.25\times t$$

The frequency calculated is indeed the resonance frequency of the antenna, which is set to operate at 2.45 GHz for all materials used (cotton, jean, and jute). These equations are necessary for calculating the key parameters, such as the impedance and resonance frequency, which are fundamental to ensuring optimal antenna performance. The roughness of the materials, based on the relative permittivity and loss tangent values, were obtained through experimental techniques like cavity perturbation and transmission line methods.

## 3. Performance Analysis of S-Parameter Characteristics

**Figure 3:**
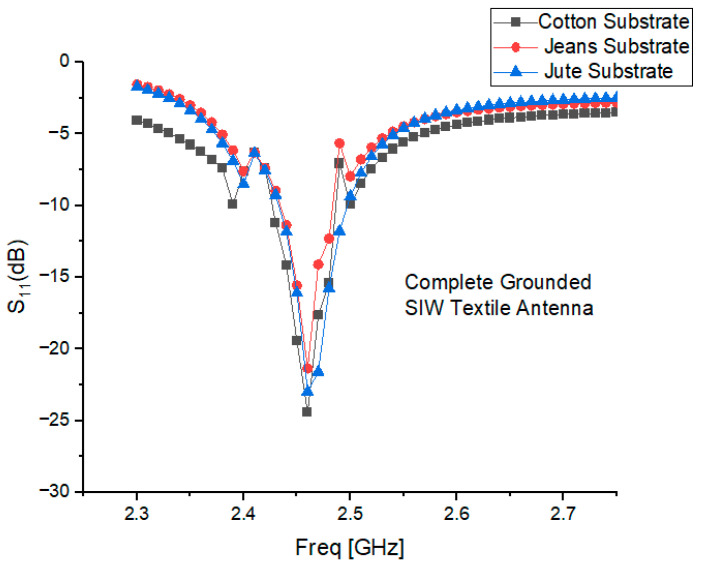
Performance analysis of S-parameter characteristics of proposed cavity-backed SIW textile antenna.

The simulations were performed across a frequency range of 1.0–3.0 GHz, with 10 MHz increments, for three cotton, jean, and jute antenna designs. The results, presented in Figure 3, reveal the following performance characteristics:

For the cotton substrate antenna, there was a resonant frequency of 2.44 GHz, a bandwidth of 110.28 MHz, and a reflection coefficient of −25.698 dB.

For the jean substrate antenna, there was a resonant frequency of 2.35 GHz, a reflection coefficient of −25.051 dB, and an impedance bandwidth of 109.61 MHz.

For the jute substrate antenna, there was a resonant frequency of 2.33 GHz, a reflection coefficient of −23.002 dB, and an impedance bandwidth of 106.71 MHz.

Variations in substrate thickness, along with inconsistencies in the dielectric properties of materials such as cotton, jean, and jute, can result in shifts in the resonant frequency.

**Figure 4:**
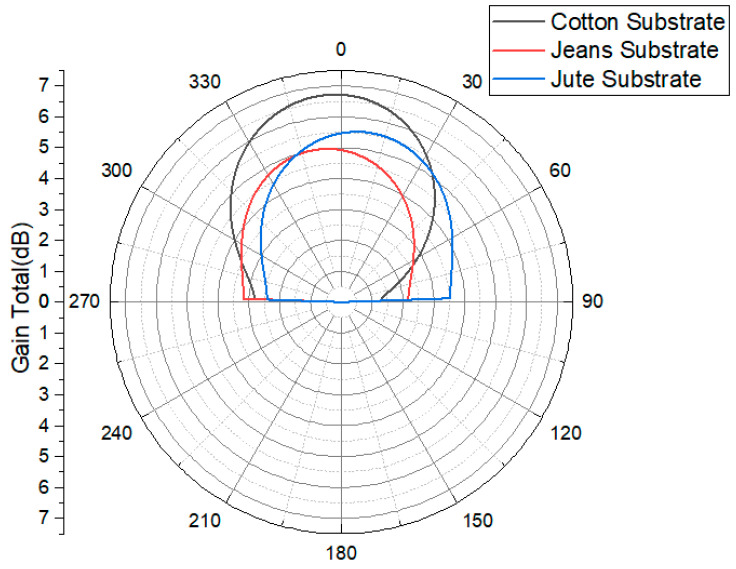
Performance analysis (gain) of cavity-backed SIW textile antenna.

The far-field radiation patterns of the three antennas, presented in Figure 4, were evaluated at their respective resonant frequencies, enabling an assessment of their performance across various frequency spectra.

The simulated radiation parameters for the three antennas are presented in Table 2. The jute antenna exhibits remarkable consistency, with a minimal deviation of 0.5% in resonant frequency. The cotton antenna demonstrates a low discrepancy of 0.82% between the simulated and theoretical values, followed by the jean fabric antenna with a 1.53% deviation. Simulated gains for cotton, jean, and jute antennas at a 2.3–2.45 GHz resonant frequency are 5.72 dBi, 5.56 dBi, and 5.56 dBi respectively.

Regarding the enhanced simulated impedance bandwidth compared to theoretical predictions, this improvement is likely due to the inductance introduced by the microstrip line feed, which facilitates better impedance matching and broadens the bandwidth. Notably, the achieved bandwidth exceeds the 150 MHz ISM band requirement, underscoring the suitability of these antennas for rapid design and deployment in wearable applications.

## 4. Artificial Magnetic Conductor (AMC) Surfaces Design

The proposed antenna is placed on a metal backing (AMC), aiming to minimize the amount of body radiation absorbed and to enhance the antenna’s ability to focus signals [24]. The proposed AMC unit cell is designed as a circular patch structure to enhance antenna performance by ensuring in-phase reflection within the 2.3–2.45 GHz range. When the antenna operates at its peak frequency, these backings act like an inductor–capacitor (L-C) tank circuit that serves to create AMC.

**Figure 5:**
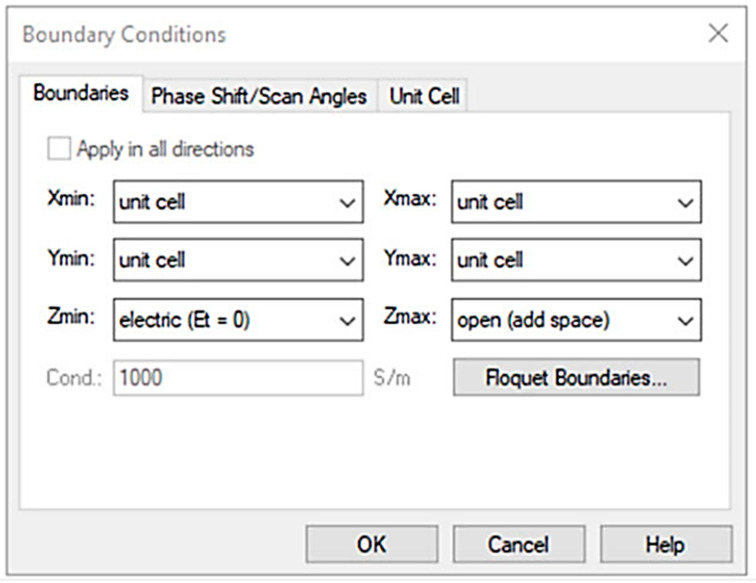
Boundary conditions for in-phase reflection characteristics.

**Figure 6:**
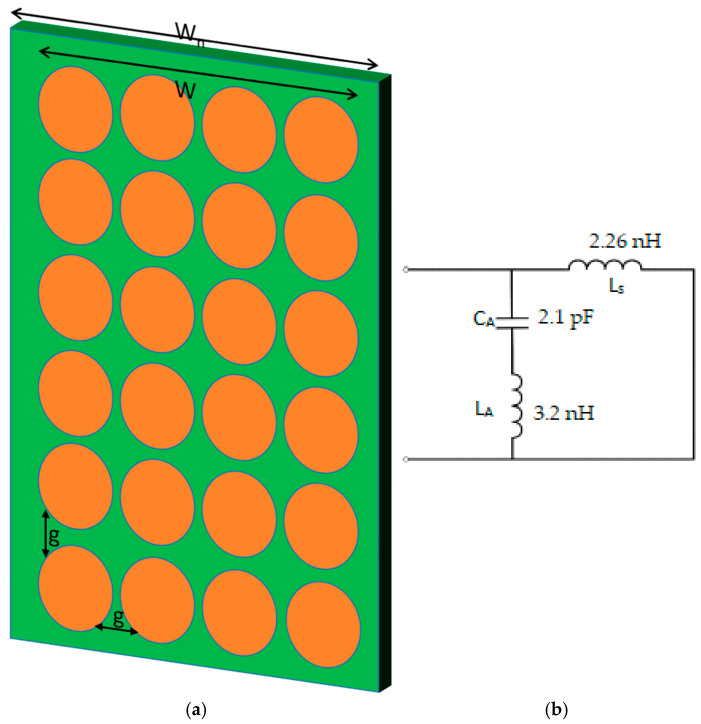
(**a**) Geometry of circular-shaped patch-type AMC array cell. (**b**) Equivalent circuit.

The key purpose of the recommended AMC backing is to ensure that the reflected wave is in phase with the incident wave at the antenna’s peak frequency of 2.45 GHz, resulting in improved antenna efficiency and gain. When the antenna is installed on a metal ground plane at a distance of less than λ/4, the destructive interference of the reflection phase (180° to the forward radiation) degrades the performance and reduces the total efficiency. However, the AMC structure’s 0° reflection phase enables constructive interference between the original and image currents, even at a distance significantly smaller than λ/4, leading to better performance and radiation efficiency. Figure 5 depicts the boundary conditions and simulation setup used for the in-phase characterization of the proposed AMC structure under a normal incident plane wave. The AMC is made up of a 4 × 6 circular-shaped patch-type array of unit cells [26], covering a total dimension of 5 × 7 cm^2^. Figure 6a,b show the geometrical design of the designed circular-shaped patch-type AMC array cell and the equivalent circuit model of the proposed AMC structure respectively. In this series LC circuit, the inductance (L_A_) represents the metal conductor in the loop and can be calculated using the following relation

(5) $$L_{A}=\ln\frac{\mu_{0}}{4\pi }\ln\left\{1+\frac{{32h}_{s}^{2}}{w_{n}^{2}}\right.\left.\left[1+\sqrt{\left.1+{\left(\left.\frac{\pi w_{n}^{2}}{{8h}_{s}^{2}}\right)\right.}^{2}\right]}\right.\right\}$$

Due to the gap (g) between the horizontal conductors of the neighboring unit cells, a capacitance (C_A_) arises, and its value is determined by the equation:

(6) $$C_{A}=\frac{W\varepsilon_{0}(1+\varepsilon_{r})}{\pi }\cosh^{-1}\left(\frac{W+g}{g}\right)$$

Inductance L_s_ represents the load inductance and can be determined using the following relation:

(7) $$L_{S}=\mu_{0}h_{s}$$

In Equations (5)–(7), the following notation is used:

μ_o_ represents the permeability of free space = 4π × 10^−7^ H/m;

ε_o_ represents the permittivity of free space = 8.854 × 10^−12^ F/m;

h_s_ represents AMC substrate thickness = 1.8 mm.

W_n_ represents the effective width of the AMC structure, which is 5 mm. W represents the width of the conductive material, which is 3 mm, and g represents the gap between the conductors, which is 0.5 mm within the unit cell.

The calculated inductance value L_A_ is approximately 3.2 nH. The calculated capacitance value C_A_ is approximately 2.1 pF. The calculated inductance L_S_ is approximately 2.26 nH for a substrate thickness of 1.8 mm.

**Figure 7:**
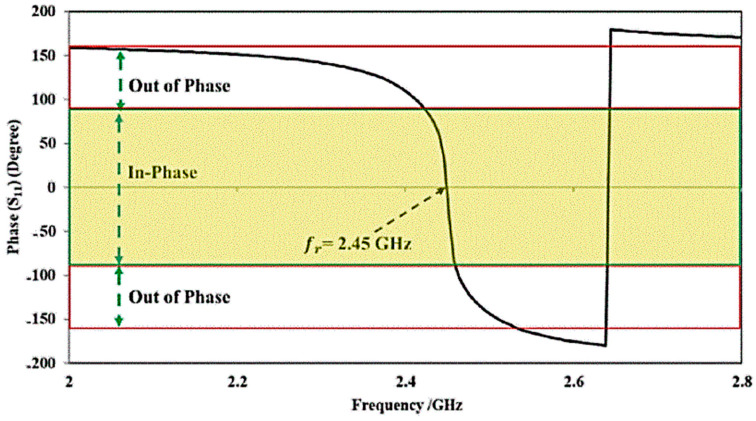
In-phase simulation of 2.45 GHz AMC unit cells with normal plane incidence.

Figure 7 shows the simulated in-phase reflection response of the proposed AMC structure at 2.45 GHz under a typical incident plane wave. Within the frequency range of 2.3–2.45 GHz, the reflection coefficient remains in phase, with the phase angle ranging between −90° and +90°, peaking at 2.45 GHz. While the backplane provides shielding from direct radiation, the AMC structure further improves performance by reducing the impedance mismatch caused by lossy human tissue, reducing the effect of impedance mismatch and maintaining optimal radiation patterns, which is particularly beneficial for wearable applications.

## 5. Antenna Integrated with AMC

**Figure 8:**
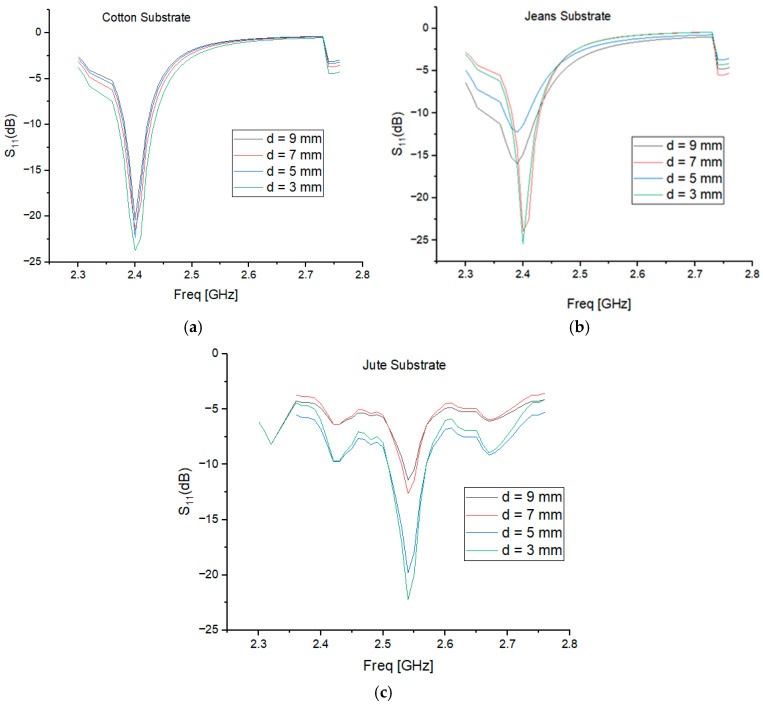
Simulated reflection coefficient of (**a**) cotton, (**b**) jean, and (**c**) jute with varying separation distances from the AMC Plane.

**Figure 9:**
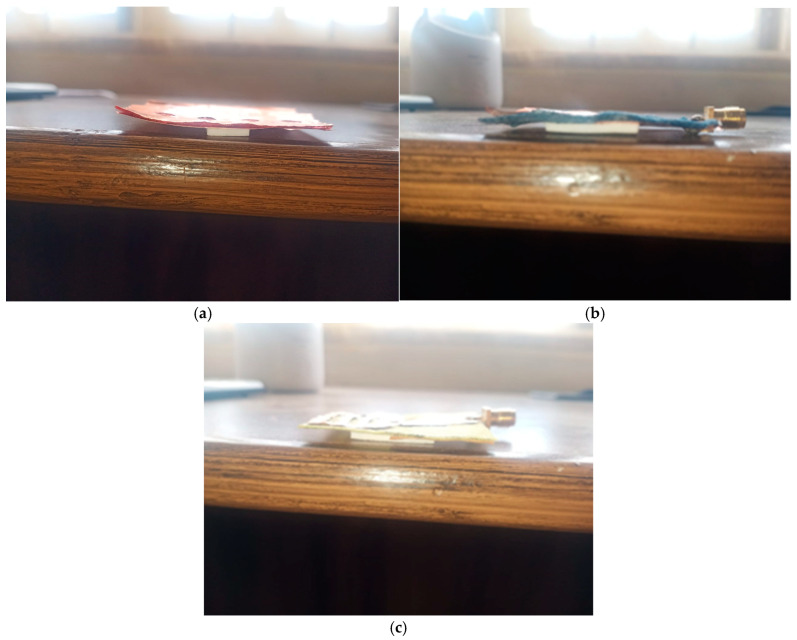
Isolation of AMC plane with textile antennas using a 3 mm thick layer of polyurethane foam. (**a**) Cotton, (**b**) jean, and (**c**) jute.

This section investigates the impact of integrating an AMC surface with an SIW textile antenna on key antenna parameters. The separation distance between the AMC surface and the antenna and the array dimensions of the AMC surface also plays a crucial role in influencing radiation patterns and gain. Several separation distances were examined, including 3 mm, 5 mm, 7 mm, and 9 mm, as well as their impact on the |S_11_| response, as depicted in Figure 8a–c. The results show that varying the separation distance has a significant effect on performance, a separation distance higher than 7 mm (0.05λo) increases the total height of the AMC-integrated design. The AMC-integrated design was, therefore, compromised between its overall size and its functionality, and a 3 mm gap was found to provide an optimal balance between gain, SAR reduction, and radiation efficiency, ensuring stable performance and user safety. To further enhance user safety and comfort, a 3 mm gap filled with a polyurethane foam layer is placed between the AMC-backed antenna and the human body, as shown in Figure 9a–c. This foam, with its low permittivity, lightweight nature, and flexibility, acts as an insulating barrier, preventing direct contact while preserving antenna performance. By reflecting more energy away from the body, the AMC surface helps reduce SAR and focuses radiation in the desired direction, thus improving antenna gain.

The differences in the |S_11_| values observed in Figure 8a–c are primarily influenced by the separation distance between the antenna and the AMC backing, as well as the dielectric properties of the substrate materials (cotton, jean, and jute). Smaller separation distances, such as 3 mm, improve impedance matching and reduce reflection losses, while larger distances lead to less effective coupling and higher reflection coefficients. Additionally, substrates with higher dielectric constants, like jean and jute, exhibit greater variations in S_11_ due to increased electromagnetic energy absorption compared to cotton. Interaction with the human body further contributes to these differences, as the high dielectric constant of tissues alters the effective permittivity and shifts the resonance frequency. These factors collectively explain the observed variations in S_11_ values across different configurations.

**Figure 10:**
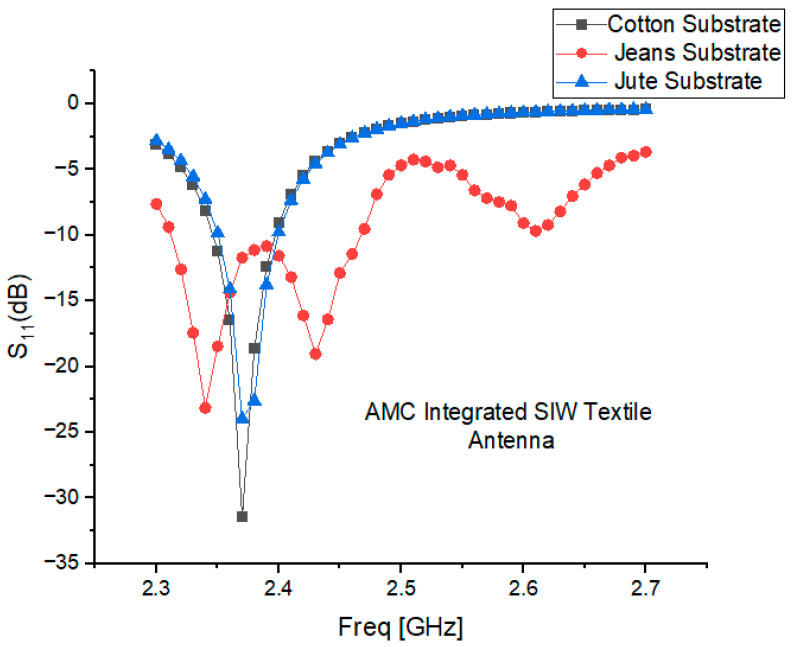
Simulated reflection coefficient characteristics of cotton, jean, and jute textile antennas separated from the AMC plane using a 3 mm layer of polyurethane foam.

A strong resonance is observed for the cotton substrate at 2.38 GHz, as shown in Figure 10. The deviations in the resonant frequencies for jean and jute are 3.23% and 1.64%, respectively. These shifts in resonance can be attributed to differences in the surface texture or weave pattern of the materials, which alter the path of electromagnetic waves.

**Figure 11:**
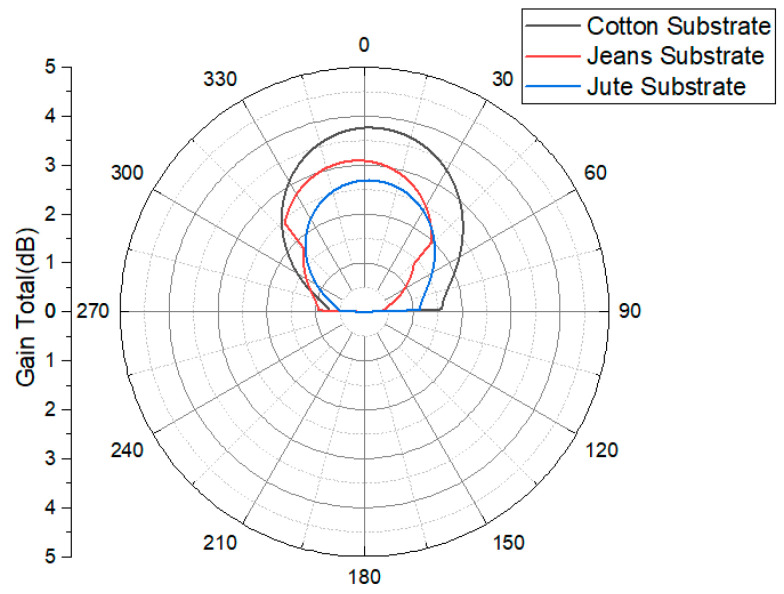
Simulated gain radiation patterns of AMC-integrated cotton, jean, and jute substrate normalized radiation patterns [dBi] at 2.45 GHz.

Figure 11 illustrates the antenna radiation patterns at a frequency of 2.45 GHz for three different substrate materials—cotton, jean, and jute—providing a visual representation of the relationship between the antenna’s radiation characteristics and the spatial angle. When the AMC structure is integrated with the antenna, the amplitude of the back lobe decreases significantly compared to the main lobe. The main lobe’s maximum radiation direction aligns with the Z-axis of the antenna, resulting in enhanced directional orientation. The inclusion of the AMC structure in the antenna design ensures that the radiation is directed toward the test subject’s body, achieving the unilateral radiation direction required for wearable devices.

The strategic integration of the AMC structure as a reflector significantly enhances the gain of the wearable cavity-backed SIW textile antenna at 2.45 GHz. Specifically, the gain increases by 1.37 dB (from 5.72 to 7.09 dBi) for cotton substrates, 1.16 dB (from 5.56 to 6.72 dBi) for jean substrates, and 1.20 dB (from 5.56 to 6.76 dBi) for jute substrates are shown are presented in Table 3. This improvement is attributed to the 4 x 6 AMC array, which elongates the propagation path for electromagnetic waves between the antenna and AMC, reducing power loss.

While the gain improvement for the cotton substrate is relatively small at 1.37 dB, this still provides meaningful benefits in body-centric environments, where even minor gains can enhance signal reliability and efficiency. Additionally, the AMC structure contributes significantly to other aspects, such as SAR reduction and impedance matching, which play crucial roles in wearable antenna performance. Larger gain improvements are observed for other substrates, like jean (1.16 dB) and jute (1.20 dB), emphasizing the material-dependent impact of the AMC structure.

## 6. Bending Analysis

The purpose of a bending analysis of wearable antennas is to understand and evaluate the performance and reliability of the antenna when it is subjected to bending or flexing due to the movements and deformations caused by the human body. Integrated into clothing or worn on the body, wearable antennas need to be capable of withstanding the mechanical stresses and strains associated with body movement. Antennas were bent in both the x and y directions to determine the impact of the bending radius on |S_11_|. Due to the unique composition of the test person’s body, the antenna clothing stretches along both axes. Various types of bending for wearable cavity-backed SIW textile antennas are investigated, focusing on two types of bending characteristics: (1) Y-axis bending, which refers to bending along the length, and (2) X-axis bending, which refers to bending along the breadth. For both scenarios, the bending angle varies between 0 and 60 degrees.

The curvature of a bent antenna is solely determined by its bending angle. Typically, the curvature is achieved by shaping the substrate of the textile antenna over a cylindrical structure with a radius Rc. To calculate the curvature of such a cylindrical structure, Equation (8) is applied in conjunction with the formula for the circumference of a circle.

(8) $$C_{R}=\frac{\left(L\times 360\right)}{\left(\theta \times 2\pi \right)}$$

where *L* is the substrate length of the antenna.

### 6.1. Y-Axis Bent over Analysis

**Figure 12:**
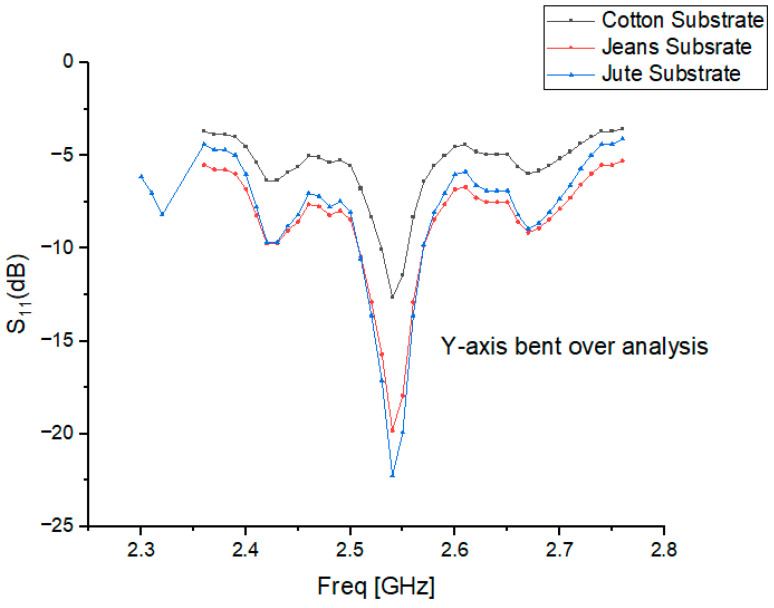
Resonant frequency and reflection coefficient for y-axis bending at 30 degrees for cotton, jean, and jute materials.

The wearable cavity-backed SIW textile antenna is capable of bending along the Y axis if it bends within its length plane. As a result of Y-axis twisting, as shown in Figure 12, at various bending angles ranging between 0 and 60 degrees with increments of 15 degrees, the designed cotton antenna is evaluated for Y-axis flexibility.

### 6.2. X-Axis Bent over Analysis

**Figure 13:**
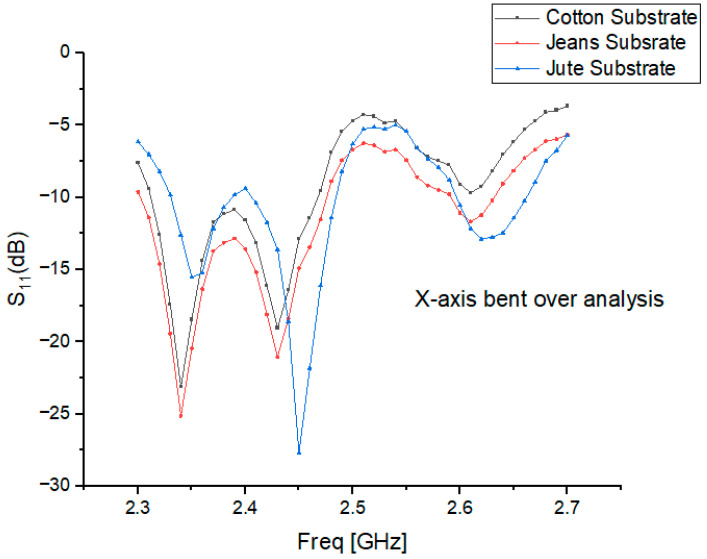
Resonant frequency and reflection coefficient for x-axis bending at 30 degrees for cotton, jean, and jute materials.

For X-axis bending, the antenna is twisted along its longitudinal plane with bending angles ranging from 0° to 60°, as shown in Figure 13. The wearable cavity-backed SIW textile antenna undergoes X-axis bending when twisted along the longitudinal plane. Various bending angles, ranging from 0 to 60 degrees, were applied to the designed wearable textile antenna to evaluate its flexibility response along the X-axis.

### 6.3. Effect of Bending Angles on Resonance Frequencies

In its flat configuration, the wearable cavity-backed SIW textile antenna operates at frequencies between 2.3 and 2.45 GHz. When subjected to bending, the resonant frequency shifts due to changes in geometry and the effective dielectric constant of the substrate. At a 15° bending angle, the resonant frequency rises to the range of 2.35–2.7 GHz, while further bending to 30° causes it to shift downward to 2.48–2.5 GHz. Beyond 60° of bending, the resonant frequency returns to higher bands, operating between 2.35 and 2.45 GHz, as depicted in Figure 12 and Figure 13.

These frequency deviations, influenced by bending angles and substrate properties, highlight the importance of robust antenna designs for wearable applications. The observed trends in Table 4 and Table 5 indicate that bending can cause either upward or downward shifts in the resonance frequency, depending on the material and bending angle. For cotton, the reflection coefficients indicate moderate losses under bending, while jean fabric exhibits higher losses due to its denser structure and greater sensitivity to bending. Similarly, jute substrates show increased reflection losses at certain angles, which is consistent with their higher dielectric constant and affects the impedance matching and efficiency.

The results emphasize the need for materials and designs that minimize frequency shifts and maintain stable impedance matching, ensuring reliable performance even under dynamic conditions. These findings align with recent studies on textile antennas, underscoring the impact of substrate properties and bending angles on antenna behavior.

## 7. Influence of Human Tissue on Antenna Radiation Characteristics

The diverse and suboptimal composition of the test subject’s body impacts the antenna’s radiation performance parameters, diminishing its effectiveness when placed close to the body. Therefore, the initial evaluation of the antenna’s capabilities is performed in free space, followed by experiments on a flat structure integrated within the test person’s body. To achieve this, a three-layered test person’s body model is designed and simulated in Ansys HFSS 13 [27]. This model comprises muscle, fat, and skin layers. The typical resistivity and permittivity of these components at a frequency of 2.4 GHz are as follows: muscle (ρ_r_ = 1.705; ε_r_ = 52.79), fat (ρ_r_ = 0.1; ε_r_ = 5.28), and skin (ρ_r_ = 5.0138; ε_r_ = 31.29). These layers have dimensions of 20 mm, 5 mm, and 2 mm, respectively. However, due to the complexity of the simulation, the moisture content in these layers has not been factored in.

The use of flexible substrates, such as cotton, jean, and jute, ensures that the antenna maintains its performance, even when worn in close proximity to the human body.

**Figure 14:**
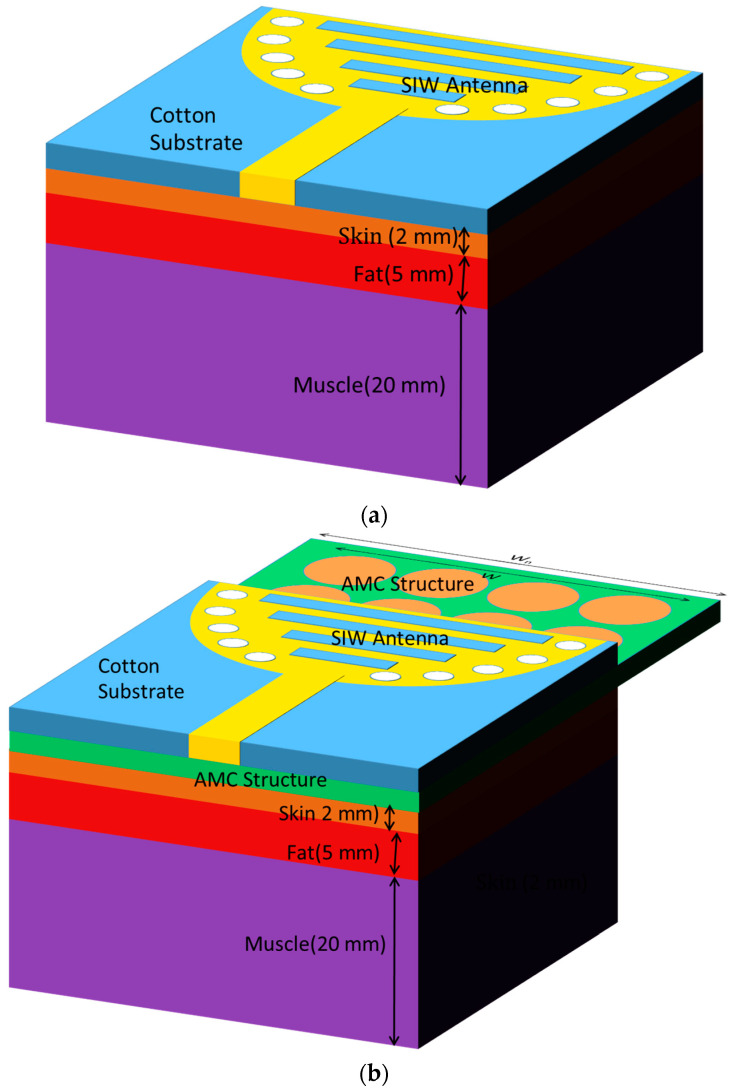
Three-layer test person’s body models were tested on (**a**) wearable antenna with cotton Substrate. (**b**) Antenna affixed on AMC structure.

**Figure 15:**
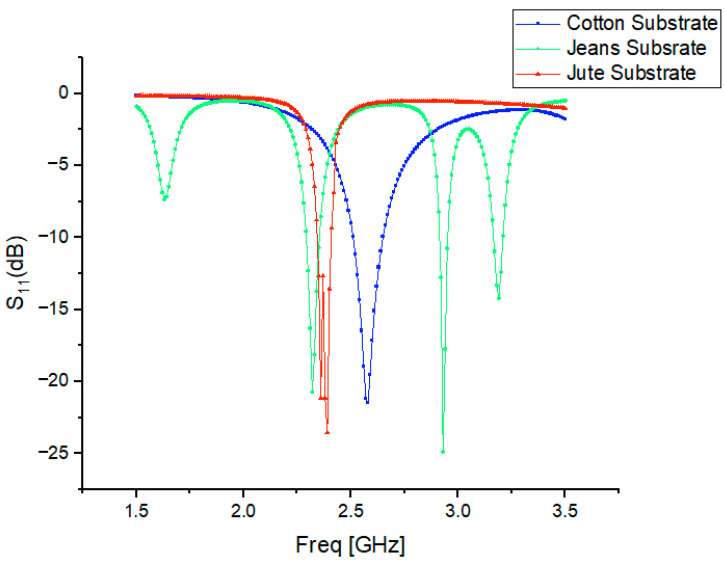
Simulated resonant frequency and reflection coefficient of all antennas affixed on three-layer test person’s body models.

A typical flat phantom model with dimensions of 50 mm × 50 mm × 27 mm was considered to emulate two-thirds of muscle tissue, as recommended for wearable antenna studies. A wearable cavity-backed SIW textile antenna was mounted on a three-layer test person’s body model illustrated in Figure 14a and a wearable antenna integrated with a three-layer test person’s body model in the presence of the AMC reflector illustrated in Figure 14b to investigate its radiation characteristics near the human body. The performances of the antennas with cotton, jean, and jute substrates were evaluated in the OFF-body state, considering key metrics such as gain, reflection coefficient, and radiation patterns. Notably, the measured reflection coefficients were −23.67 dB (cotton), −17.3 dB (jean), and −21.71 dB (jute) in the OFF condition, as illustrated in Figure 15.

In the OFF condition, the reflection coefficient of the wearable cavity-backed SIW textile antenna with substrates like cotton, jean, and jute shifts slightly to the right of the 2.45 GHz resonant frequency. However, when positioned near the human body, the high dielectric constant of body tissues, such as skin, fat, and muscle, increases the effective permittivity surrounding the antenna. This interaction causes a downward shift in the antenna’s resonant frequency, influencing its performance and lowering its reflection coefficient.

**Figure 16:**
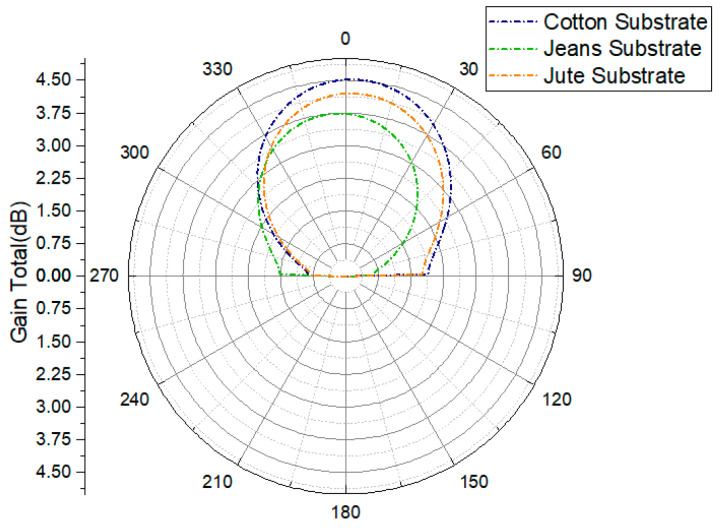
Simulated radiation patterns normalized to 0 dBi are shown for a frequency of 2.45 GHz affixed on three-layer test person’s body models.

The radiation characteristics of the antenna are significantly influenced by its placement on the body. The E-plane radiation patterns of the cotton, jean, and jute antennas, as well as those mounted on a flat structure, remain consistent with the resonance frequency, as shown in Figure 16. However, when attached to a human body model, the peak gain decreases slightly by 0.05 dB. Additionally, the radiative efficiency drops by 1% due to the higher resistivity of the skin layer, and the beam width widens from 65.6 to 81.3 degrees. These results underscore the importance of considering human body effects in the design of wearable antennas. The simulated gains at a resonant frequency of 2.45 GHz for the cotton, jean, and jute substrates are 4.36 dBi, 3.75 dBi, and 3.59 dBi, respectively, reflecting the lossy nature of the human body. This characteristic notably reduces both gain and efficiency.

## 8. Results and Discussion

**Figure 17:**
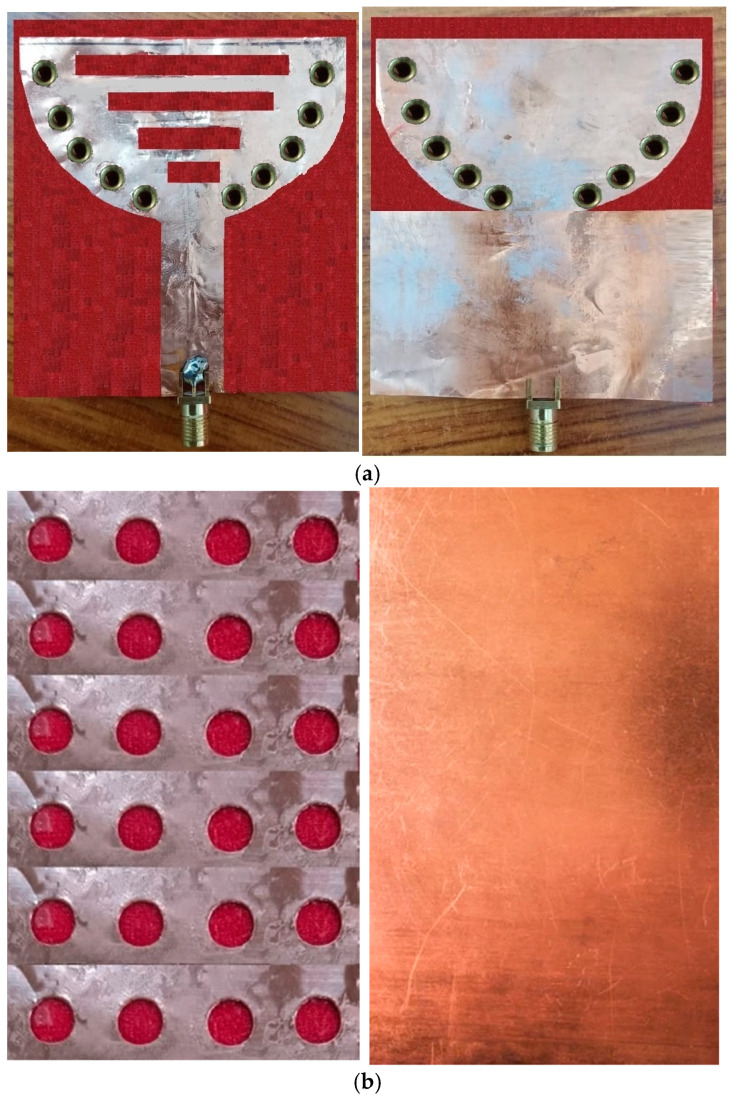
Fabricated cavity-backed SIW textile antenna and AMC reflector plane with cotton fabric (**a**) front and (**b**) back view.

**Figure 18:**
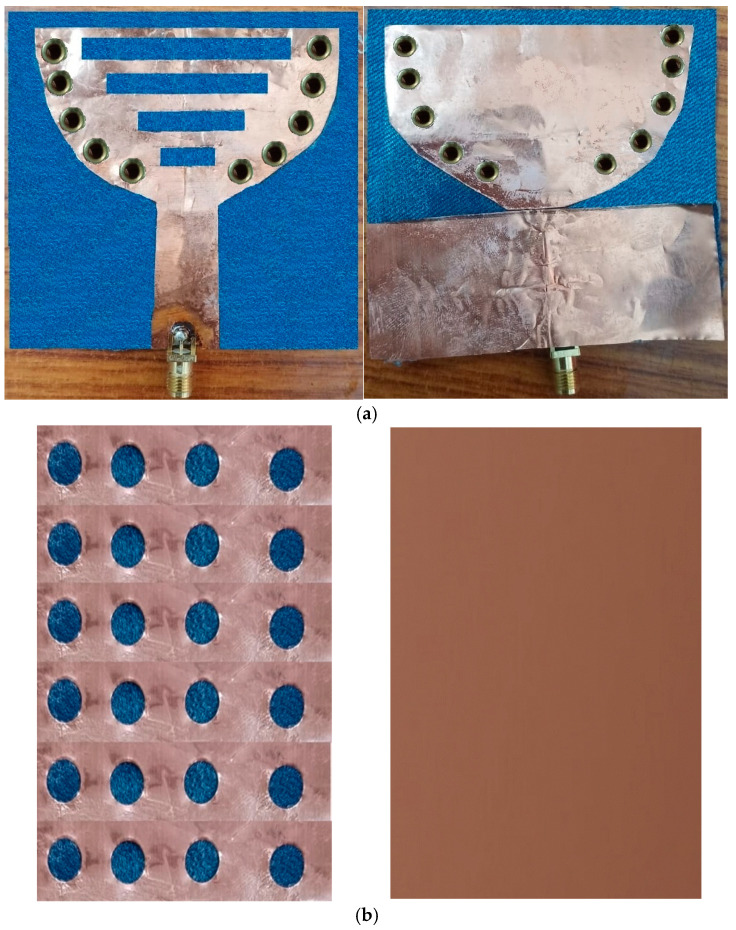
Fabricated cavity-backed SIW textile antenna and AMC reflector plane with jean fabric (**a**) front and (**b**) back view.

**Figure 19:**
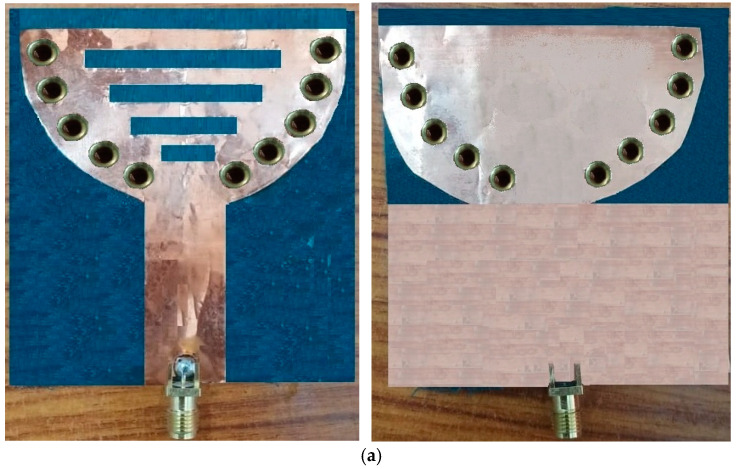

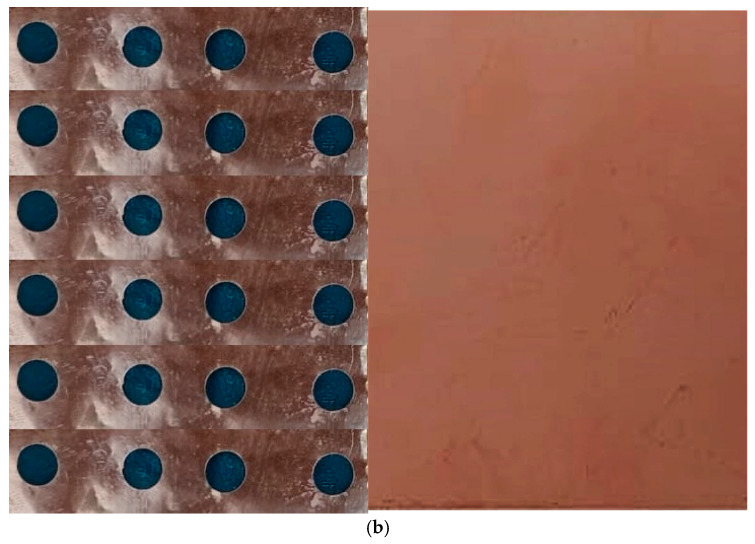
Fabricated cavity-backed SIW textile antenna and AMC reflector plane with jute fabric (**a**) front and (**b**) back view.

A novel wearable SIW textile antenna design, leveraging three non-conductive fabrics (cotton, jean, and jute) as substrates, has been developed for on-body applications. This antenna demonstrates exceptional performance across the 2.3–2.45 GHz range. To ensure precision, the SIW patch and base were precisely fabricated, with dimensional accuracy controlled within ±100 microns (±0.1 mm). The antenna’s electrical components were carefully assembled, utilizing copper sheets adhered to the fabric with tape, eliminating air gaps between the fabric and the components. The integration of AMC surfaces effectively reduces SAR values and enhances gain. Strategic adjustment of the probe’s position achieved an impedance of 50 ohms at the central frequency, resulting in optimal matching characteristics. Figure 17, Figure 18 and Figure 19 illustrate visual representations of the antennas, which demonstrate the structure of the SIW cavity. These antennas feature metallic patches on the top and bottom layers, constructed using cotton, jean, and jute fabrics, respectively, with interconnections formed by metal vias.

The performance of the cavity-backed SIW textile antenna, designed with three materials (cotton, jean, and jute) and integrated with an AMC (artificial magnetic conductor) backing, was evaluated in practical on-body tests shown. The AMC backing was positioned behind the antenna to mitigate body interference. Measurements were conducted using the Shockline MS46122B Vector Network Analyzer (VNA) [28] to assess the reflection coefficient, bandwidth, and input impedance of the prototypes.

**Figure 20:**
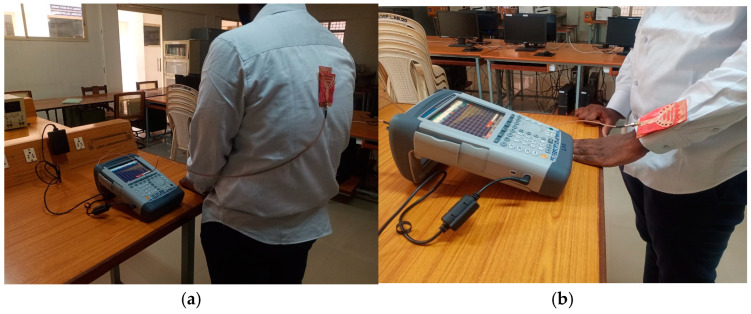
Testing setup: AMC-integrated cavity-backed SIW textile antenna with cotton fabric affixed on the body of test person. (**a**) Spine and (**b**) forelimb.

**Figure 21:**
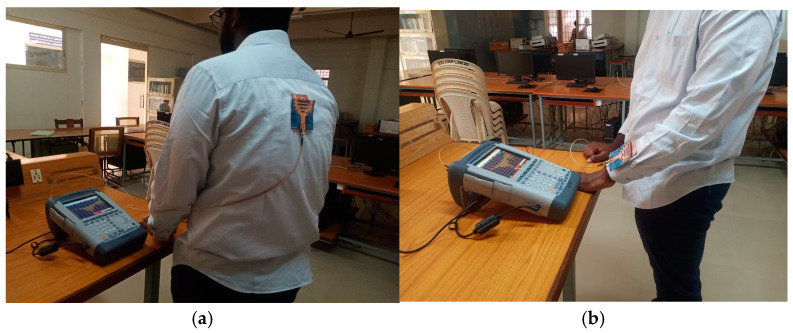
Testing setup: AMC-integrated cavity-backed SIW textile antenna with jean fabric affixed on the body of test person. (**a**) Spine and (**b**) forelimb.

**Figure 22:**
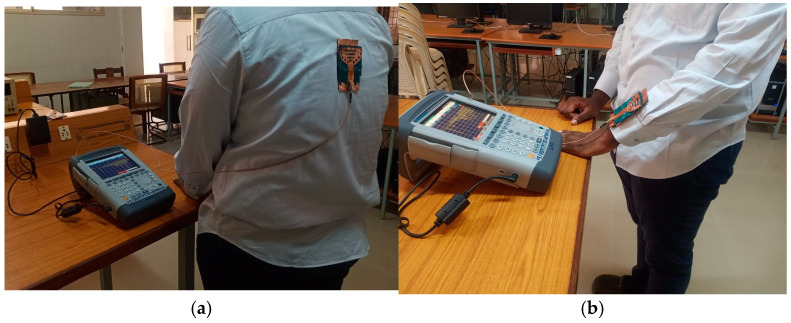
Testing setup: AMC-integrated cavity-backed SIW textile antenna with jute fabric affixed on the body of test person. (**a**) Spine and (**b**) forelimb.

**Figure 23:**
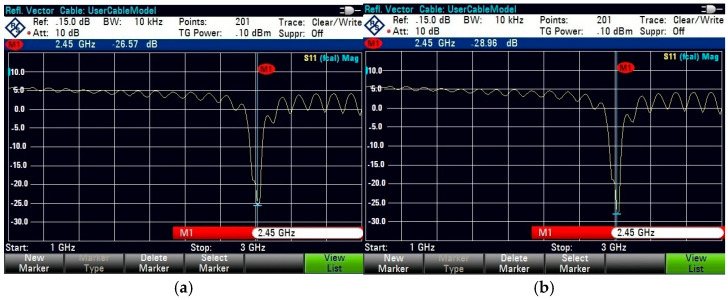
Snapshot of reflection coefficient S_11_ (dB) measurement using VNA for the AMC-supported cotton antenna placed close to a test person’s (**a**) human spine. (**b**) Forelimb of radius 50 mm.

**Figure 24:**
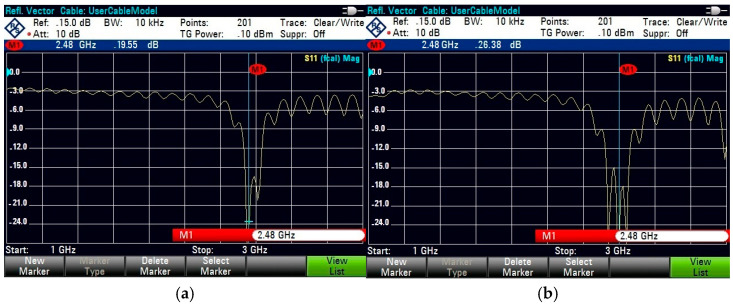
Snapshot of reflection coefficient S_11_ (dB) measurement using VNA for the AMC-supported jean antenna placed close to a test person’s (**a**) human spine. (**b**) Forelimb of radius 50 mm.

**Figure 25:**
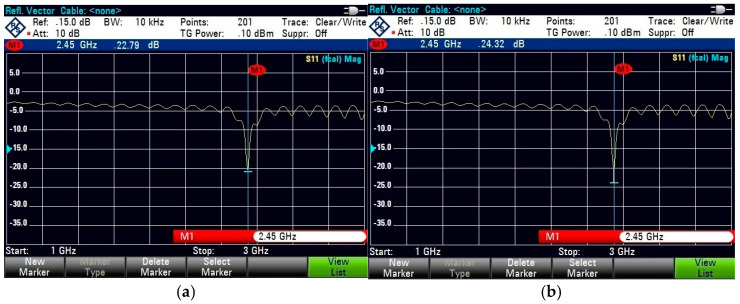
Snapshot of reflection coefficient S_11_ (dB) measurement using VNA for the AMC-supported jute antenna placed close to a test person’s (**a**) human spine. (**b**) Forelimb of radius 50 mm.

For on-body testing, the antenna prototypes were separated from the AMC surface by a 3 mm layer of polyurethane foam and placed on the human spine and forelimb, as shown in Figure 20, Figure 21 and Figure 22. The AMC-backed cavity-supported SIW textile antennas demonstrated excellent performance. The cotton substrate achieved reflection coefficients of −26.56 dB on the spine and −28.96 dB on the forelimb at 2.45 GHz (Figure 23). The jean substrate recorded values of −19.55 dB and −26.38 dB at 2.48 GHz (Figure 24), while the jute substrate showed −22.78 dB and −24.32 dB at 2.45 GHz (Figure 25).

The inclusion of AMC significantly enhanced impedance matching, reducing the detuning effects caused by proximity to the body. This improvement resulted in a broader −10 dB bandwidth of 150 MHz (2.32–2.48 GHz), demonstrating superior performance in wearable antenna applications.

**Figure 26:**
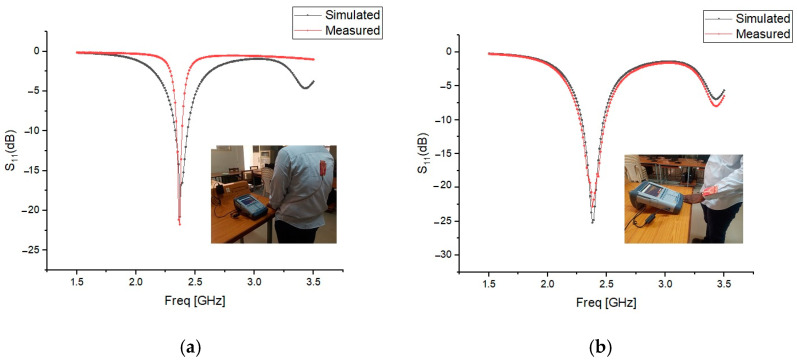
Reflection coefficient S_11_ (dB) of the AMC-supported cotton antenna placed close to a test person’s (**a**) human spine. (**b**) Forelimb of radius 50 mm. Solid red line: measured results. Solid black line: simulated results.

**Figure 27:**
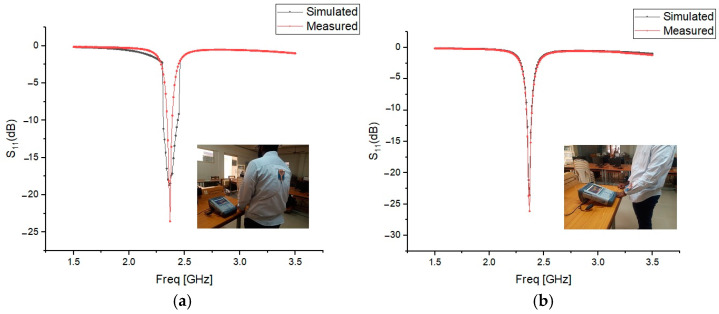
Reflection coefficient S_11_ (dB) of the AMC-supported jean antenna placed close to a test person’s (**a**) human spine. (**b**) Forelimb of radius 50 mm. Solid red line: measured results. Solid black line: simulated results.

**Figure 28:**
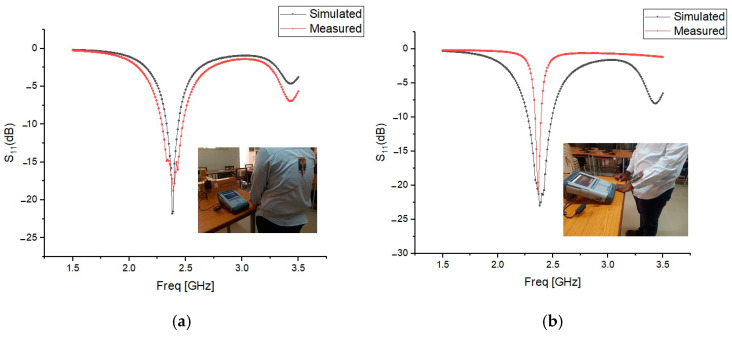
Reflection coefficient S_11_ (dB) of the AMC-supported jute antenna placed close to a test person’s (**a**) human spine. (**b**) Forelimb of radius 50 mm. Solid red line: measured results. Solid black line: simulated results.

The presence of the human body slightly degraded the radiation pattern, reducing the maximum radiated power and efficiency. However, the AMC enhancements improved gain and radiation efficiency, demonstrating the benefits of using AMC structures. Figure 26, Figure 27 and Figure 28 validate the consistency between the reflection coefficient S_11_ of the simulation and experimental results, confirming the effectiveness of the AMC-enhanced cavity-backed SIW textile antenna.

This study successfully integrates cavity-backed SIW textile antenna technology at 2.45 GHz with flexible textile substrates, showcasing significant improvements in performance and safety through AMC enhancements. The results pave the way for next-generation wearable antennas with improved functionality and user safety.

**Figure 29:**
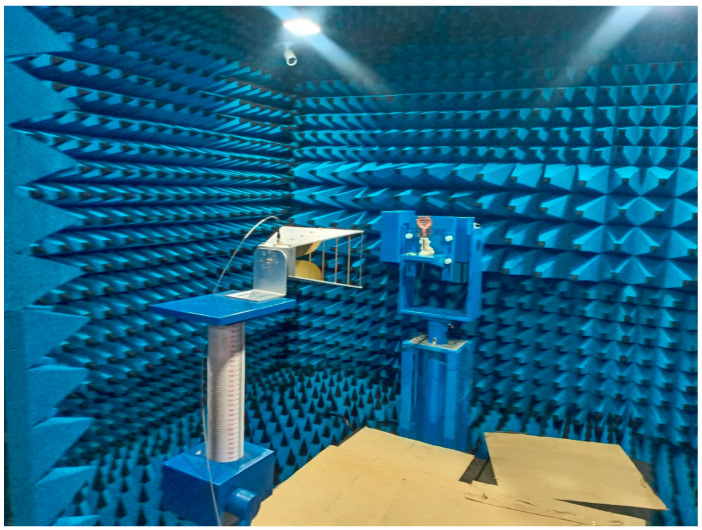
Measurement setup of cotton textile antenna in a microwave-shielded far-field anechoic chamber.

**Figure 30:**
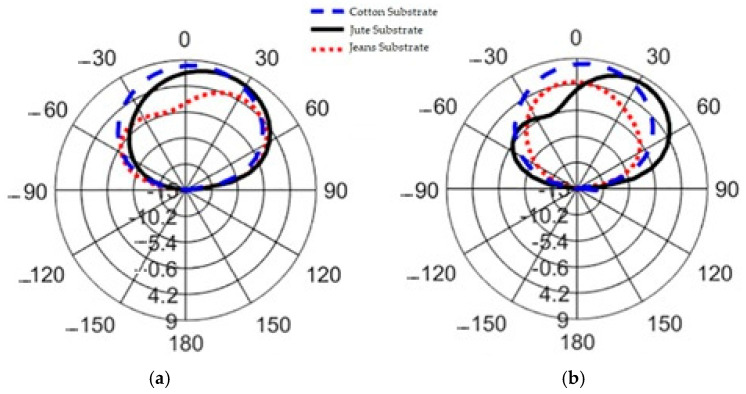
Tested radiation patterns (degree vs. dB) of an antenna placed close to a test person’s (**a**) human spine. (**b**) Forelimb of radius 50 mm.

Figure 29 illustrates the measurement setup for evaluating the proposed antenna’s performance in a microwave-shielded far-field anechoic chamber, ensuring an accurate assessment of its reflection characteristics and radiation characteristics. The configuration includes a standard gain horn antenna positioned 2.5 m from the device under test (DUT) with horizontal polarization, and an Shockline MS46122B Vector Network Analyzer (VNA) was used for S_11_ parameter and gain radiation pattern measurements. Figure 30 shows the radiation patterns when the antenna is positioned near the test subject’s spine and forelimb with a radius of 50 mm.

At 2.45 GHz, the E-plane radiation patterns (φ = 0°) of the cotton-, jean-, and jute-based antennas were analyzed. The measured gains were slightly lower than expected, with cotton achieving 5.6 dBi (expected: 7.09 dBi), jean reaching 4.9 dBi (expected: 6.72 dBi), and jute attaining 5.2 dBi (expected: 6.76 dBi). These deviations are primarily due to the human body’s absorption of radiated power, along with factors such as fabrication tolerances, environmental conditions (e.g., room reflections and temperature), and substrate material properties like permittivity and loss tangent. Impedance mismatches in real-world designs compared to ideal simulations also contributed to the differences. A minor discrepancy was observed between the measured and simulated far-field off-body gains, as summarized in Table 5, which highlights the superior performance of the cotton-based wearable antenna compared to jean and jute

The simulated radiation efficiency was 80%, while the actual measured efficiency decreased to 70% due to these factors. Cotton outperformed jean and jute as an antenna substrate thanks to its low loss tangent and moderate dielectric constant, which minimize energy loss and balance absorption with radiation efficiency. In contrast, jean and jute exhibited higher energy absorption due to their higher dielectric constants and loss tangents. Despite these variations, all performance metrics fell within acceptable ranges, highlighting the antenna’s suitability for wearable applications.

## 9. SAR Analysis Overview

This section examines how wearable cavity-backed SIW textile antennas (SIW TAs) interact with the test person’s body, with a specific focus on their SAR. The limits for SAR, as set by the International Electro-Technical Commission (IEEE) through IEEE/IEC 62704 [29] were considered in this analysis. The SAR values were measured for both 1 g and 10 g of tissue, with the latter showing a higher SAR limit of 2 W/kg, which is in alignment with European safety standards (ICNIRP) [30]. It is important to note that traditional antennas tend to experience frequency shifts when placed near the test person’s body, leading to impedance mismatches that increase the reflected power and, consequently, the SAR. In this study, materials such as cotton, jean, and jute were used. The physical flexibility of cotton and the presence of air gaps in the fabric contribute to its ability to absorb reflected radiation, thus reducing its impact on the test person’s body.

### SAR Investigation of Textile Antennas

**Figure 31:**
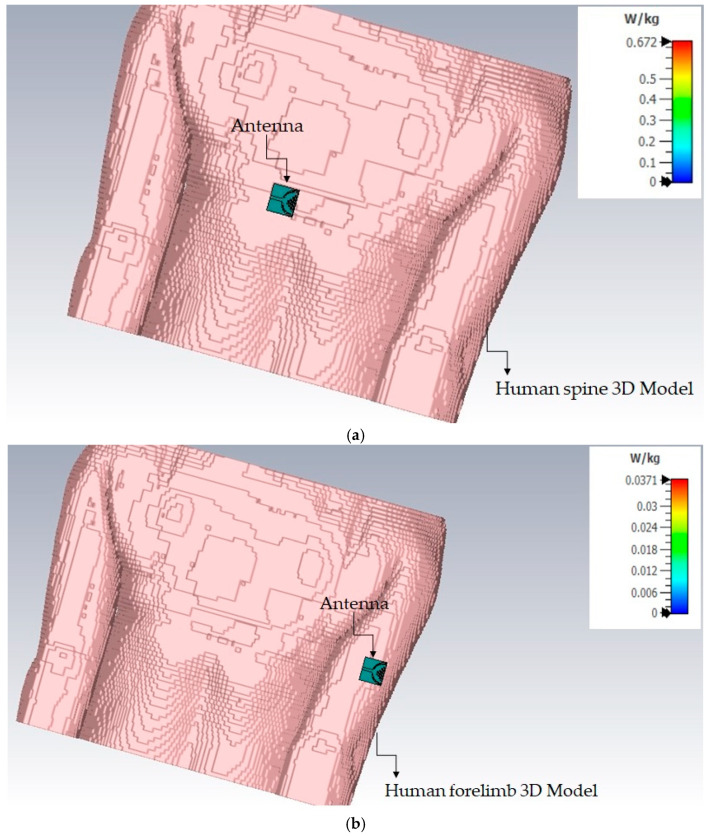
SAR analysis of SIW wearable antennas made of cotton, jean, and jute fabrics on (**a**) human spine and (**b**) forelimb at 2.45 GHz using 3-layer body phantoms with a mass of 10 g.

An SAR analysis was performed using CST Microwave Studio (Version S2, 2023) [31] using the IEEE C95.3 averaging method [32]. The study presents an overview of the recommended SAR values for the cotton smart textile antenna analyzed within the 2.3–2.45 GHz range, with a center frequency of 2.45 GHz, reducing the SAR by 0.672 W/kg on the human spine and 0.341 W/kg on the human forelimbs. The jean substrate has a maximum SAR of 0.762 and 0.698 W/kg for the human spine and forelimb. The jute material has a maximum SAR of 0.730 W/kg for the human spine and 0.386 W/kg for the human forelimb, as shown in Figure 31, indicating that it complied with and is safe to use within the standards established by the Federal Communications Commission (FCC) and the International Commission on Non-Ionizing Radiation Protection (ICNIRP). These findings suggest that the wearable IoT technology using these smart textile antennas operates safely across a range of tissue weights.

The cotton substrate demonstrates the highest performance among the three materials, with a reflection coefficient (S_11_) of −26.56 dB on the spine and −28.96 dB on the forelimb, (Figure 24). Jean and jute substrates exhibit slightly lower reflection coefficients of −19.55 dB and −26.38 dB for jean (Figure 25) and −22.78 dB and −24.32 dB for jute (Figure 26) due to their higher dielectric constants, which increase electromagnetic energy absorption. The integration of AMC surfaces significantly reduces the SAR values across all materials. Cotton achieves the lowest SAR at 0.672 W/kg on the spine and 0.341 W/kg on the forelimb, making it the safest for wearable applications.

As shown in Table 5, the gain for the cotton-based antenna is 5.6 dBi on the spine and 4.5 dBi on the forelimb. In comparison, jean and jute exhibit slightly lower gains of 4.9 dBi and 3.7 dBi (jean), and 5.2 dBi and 4.2 dBi (jute), respectively. Testing on the body results in a reduction of approximately 2.24 dBi in gain and a 10% drop in efficiency, which is expected due to the lossy nature of human tissues. Despite these losses, the cotton-based antenna demonstrates strong performance, making it suitable for body-centric communication networks. Additionally, SAR values for all substrates remain well within FCC and ICNIRP safety limits, ensuring prolonged safe usage.

AMC surfaces play a crucial role in enhancing antenna performance by improving impedance matching and directing radiation away from the body. The gain improvements range from 1.16 dB for jean to 1.37 dB for cotton, while the SAR values are reduced by up to 30% across all substrates (Table 6). These improvements are essential for ensuring reliable performance and user safety in wearable applications. The alignment between the simulated and measured results confirms the robustness of the cavity-backed SIW textile antenna design under real-world conditions.

Table 7 highlights the superior performance of the proposed wearable cavity-backed SIW textile antenna with AMC backing compared to previous designs. The proposed antenna, using cotton as the substrate, achieves a gain of 5.6 dBi on the spine and 4.5 dBi on the forelimb, along with SAR values of 0.672 W/kg and 0.341 W/kg, respectively. These results are significantly better than those of the traditional designs that utilize rigid substrates or less efficient textile materials.

Compared to previous works, the proposed antenna offers advantages in terms of compact size (53 × 51 × 0.8 mm^3^ for cotton) and improved safety metrics, as it maintains low SAR values while ensuring high gain and efficiency. The incorporation of AMC surfaces further enhances the gain and reduces the SAR values, making the design suitable for wearable applications. These improvements demonstrate the practical viability of the proposed antenna for body-centric networks, offering enhanced user safety and communication reliability.

## 10. Conclusions

This study introduces a novel wearable cavity-backed SIW textile antenna augmented with artificial magnetic conductor (AMC) structures that are designed for body-centric applications. By leveraging three textile substrates—cotton, jean, and jute—the proposed design addresses key challenges in wearable antennas, including SAR reduction, gain enhancement, and impedance matching. The AMC-backed design demonstrated superior performance, with reflection coefficients of −26.56 dB (spine) and −28.96 dB (forelimb) for cotton, −19.55 dB and −26.38 dB for jean, and −22.78 dB and −24.32 dB for jute. Gain improvements were observed, reaching 7.09 dBi for cotton, 6.72 dBi for jean, and 6.76 dBi for jute, while the SAR values were significantly reduced to 0.672 W/kg (spine) and 0.341 W/kg (forelimb) for cotton, with similar reductions for jean and jute, all within international safety limits. In contrast, without AMC integration, gains were lower, at 5.72 dBi, 5.56 dBi, and 5.56 dBi for cotton, jean, and jute, respectively, with higher SAR values (e.g., 1.12 W/kg for cotton) and limited back-radiation mitigation. The inclusion of AMC notably improved antenna performance by mitigating the back radiation and enhancing the radiation efficiency, with gain increases of up to 1.37 dB for cotton alongside substantial SAR reductions. These enhancements affirm the efficacy of AMC integration in wearable designs, emphasizing its critical role in achieving safety and performance benchmarks.

Comprehensive analysis, including simulation and on-body testing, validated the antenna’s reliability under dynamic conditions, such as bending and human proximity. The incorporation of polyurethane foam as an isolating layer ensured stable performance, further reducing body-induced detuning effects. Among the substrates tested, cotton demonstrated the best overall performance, making it a strong candidate for real-world wearable applications.

The proposed design effectively bridges the gap between flexibility, safety, and high-performance metrics, marking a notable improvement over existing designs. These findings establish a robust foundation for future research and development in wearable antenna systems for wireless communication, IoT, and healthcare applications. The results highlight the potential for multi-band or wideband adaptations, integration with energy-harvesting mechanisms, and extended testing across diverse user demographics and environments, paving the way for next-generation wearable technologies.

## 11. Future Scope

This research serves as a foundational step toward developing high-performance wearable antennas, bridging the gap between comfort, safety, and advanced electromagnetic performance. The proposed design paves the way for next-generation innovations in the field of wearable technology.

- Explore advanced hybrid textile materials with enhanced dielectric properties and mechanical robustness to further optimize antenna performance under dynamic conditions;
- The antenna’s design can be adapted for multi-band or wide-band applications, facilitating its integration into emerging 5G and IoT networks;
- Extended on-body testing across diverse user demographics and environmental conditions would provide deeper insight into the design’s versatility and adaptability;
- Incorporating energy-harvesting mechanisms or sensors into the antenna structure could unlock multifunctional capabilities, enhancing its utility in smart textiles and biomedical applications.

## Figures and Tables

**Table 1:** Parametrical dimensions of proposed antenna geometry for various substrate materials.

Antenna Design Parameter	Cotton	Jean	Jute
Frequency of Operation (GHz)	2.3–2.45	2.3–2.45	2.3–2.45
Dielectric constant of the substrate	1.6	1.7	1.87
Thickness of substrate (mm)	0.8	1.1	1.2
Substrate’s loss Tangent	0.04	0.025	0.052
Ground Plane Material and SIW Patch Material	copper	copper	copper
$\mathrm{Length}\;\mathrm{of}\;\mathrm{the}\;\mathrm{substrate}\;L_{eff}$ (cm)	5.7	5.3	5.2
$\mathrm{Width}\;\mathrm{of}\;\mathrm{the}\;\mathrm{substrate}\;W_{eff}$ (cm)	5.1	5	5.1
Length of the slot Ls_1_ (cm)	2.65	2.6	2.85
Width of the slot is Ws_1_ (cm)	0.64	0.63	0.64
Length of the slot Ls_2_ (cm)	1.77	1.73	1.9
Width of the slot is Ws_2_ (cm)	0.64	0.63	0.64
Length of the slot Ls_3_ (cm)	1.32	1.3	1.42
Width of the slot is Ws_3_ (cm)	0.64	0.63	0.64
Length of the slot Ls_4_ (cm)	1.06	1.04	1.14
Width of the slot is Ws_4_ (cm)	0.64	0.63	0.64
Length of the microstrip feedline L_MS_ (cm)	2.44	2.44	2.44
Width of the microstrip feedline W_MS_ (cm)	1.15	1.15	1.15
Length of the ground plane L_g_ (cm)	5.3	5.2	5.7
Width of the ground plane is W_g_ (cm)	2.55	2.5	2.55

**Table 2:** Simulated comparative analysis of various characteristic parameters in alignment with various substrate materials.

Antenna Type	Resonant Frequency (GHz)	Reflection Coefficient (dB)	Impedance Bandwidth (MHz)
Cotton	2.44	−25.698	110.28
Jean	2.35	−25.051	109.61
Jute	2.33	−23.002	106.71

**Table 3:** Parametric analysis of cotton, jean, and jute textile antennas separated from the AMC plane using a 3 mm layer of polyurethane foam.

Antenna Parameter	Cotton	Jean	Jute
Gain (dBi)	7.09	6.72	6.76
Radiation Efficiency (%)	71.8	68.8	70.3

**Table 4:** Comparative analysis of resonance frequencies and reflection coefficients for different bending angles (Y-axis).

Bending Angle (Degrees)	Cotton (GHz)	Reflection Coefficient (dB)	Jean (GHz)	Reflection Coefficient (dB)	Jute (GHz)	Reflection Coefficient (dB)
0° (Flat)	2.30–2.45	−20.5	2.38–2.47	−22.0	2.40–2.45	−21.0
15°	2.35–2.60	−25.2	2.43–2.36	−20.5	2.43–2.39	−25.0
30°	2.48–2.50	−22.5	2.41–2.35	−27.5	2.42–2.37	−21.5
45°	2.39–2.40	−23.3	2.34–2.39	−15.3	2.35–2.41	−14.8
60°	2.41–2.43	−18.5	2.36–2.41	−14.7	2.37–2.40	−19.2

**Table 5:** Comparative analysis of resonance frequencies and reflection coefficients for different bending angles (X-axis).

Bending Angle (Degrees)	Cotton (GHz)	Reflection Coefficient (dB)	Jean (GHz)	Reflection Coefficient (dB)	Jute (GHz)	Reflection Coefficient (dB)
0° (Flat)	2.30–2.38	−20.5	2.38–2.47	−22.0	2.40–2.45	−21.0
15°	2.33–2.38	−18.7	2.37–2.43	−20.5	2.39–2.43	−19.5
30°	2.32–2.39	−9.7	2.35–2.41	−25.1	2.37–2.42	−27.5
45°	2.39–2.40	−23.3	2.34–2.39	−15.3	2.35–2.41	−14.8
60°	2.42–2.46	−12.5	2.36–2.40	−19.8	2.38–2.42	−17.5

**Table 6:** Performance analysis of the proposed textile antenna design (S_11_, gain, bandwidth, and SAR) with AMC backing.

Antenna Type	Resonant Frequency (GHz)	Reflection Coefficient S_11_ (dB)		Gain (dBi)		Impedance Bandwidth (MHz)		SAR (W/Kg)	
		Human Spine	Human Forelimb	Human Spine	Human Forelimb	Human Spine	Human Forelimb	Human Spine	Human Forelimb
Cotton	2.45	−26.56	−28.96	5.6	4.5	105.7	105.2	0.672	0.341
Jean	2.48	−19.55	−26.38	4.9	3.7	109.61	106.5	0.762	0.698
Jute	2.45	−22.78	−24.32	5.2	4.2	106.71	104.3	0.730	0.386

**Table 7:** Comparing the designed antenna with previous antenna solutions.

Ref.	Frequency (GHz)	Antenna Size (mm^2^)	Antenna Substrate/Material (mm)	Reflector-Back Antenna’s Gain (dBi)	SAR (W/Kg)
[5]	4.8	27 × 34	Pellon/1.8	6.12	1.18/0.37
[12]	2.45	68 × 38	Rogers 5880/1.57	6.88	0.244
[13]	2.4	30 × 20	denim material/0.7	7.8	0.013
[14]	1.8/2.45	124 × 90	Jean fabric/1	–	0.024/0.016
[15]	2.4	135 × 135	Polyester/0.1	8.5	0.07
[16]	2.45	46 × 46	Adopts denim/1	6.75	0.5
[17]	2.45/3.3	89 × 83	RO3003/1.52	6.2/3	0.29/0.29
[18]	2.4	50 × 50	Latex/1	0.12	0.714
[20]	2.4/5.8	–	Felt/2	–	–
[22]	2.45	–	Felt/3	2.22	0.0721
[23]	2.45	32 × 57	Pellon/3.6	4.6	0.166
[24]	2.45	30 × 45	Kapton polyim-ide/0.057	4.8	0.683
[25]	2.65	35.25 × 17.47	Adopt/1.5	2.99	1.25
[26]	2.45/1.57	85.5 × 85.5	Kevlar/5.62	1.94/1.98	0.78
This work	2.45	57 × 51 × 0.8	Cotton	5.6 (spine) 4.5 (forelimb)	0.672/0.341
	2.45	53 × 50 × 1.1	Jean	4.9 (spine) 3.7 (forelimb)	0.762/0.698
	2.45	52 × 51 × 1.2	Jute	5.2 (spine) 4.2 (forelimb)	0.730/0.386

## Data Availability

The original contributions presented in this study are included in the article. Further inquiries can be directed to the corresponding author.
